# Air Nicotine Monitoring Study at Chennai, Tamil Nadu to Assess the Level of Exposure to Second Hand Smoke in Public Places

**DOI:** 10.4103/0970-0218.62577

**Published:** 2010-01

**Authors:** TS Selvavinayagam

**Affiliations:** Health Officer and State Tobacco Control Officer, Office of Director of Public Health and Preventive Medicine, Government of Tamilnadu 359, Anna Salai, Chennai - 600 006, Tamil Nadu, India

## Introduction

Tobacco is the leading preventable cause of death in the world. It causes 1 in 10 deaths among adults worldwide. In 2005, tobacco caused 5.4 million deaths, or an average of one death every 6 s. At the current rate, the death toll is projected to reach more than 8 million annually by 2030 and a total of up to one billion deaths in the 21^st^ century as per WHO Report on the Global Tobacco Epidemic, 2008.(1)

Second-hand tobacco smoke is equally dangerous to health. It causes cancer, heart disease, and many other serious diseases affecting almost entire human body. Almost half of the world's children breathe air polluted by tobacco smoke, which worsens their asthma conditions and causes dangerous diseases. At least 200,000 workers die every year due to exposure to second-hand smoke at work.(2)

As per world health statistics 2008 report,(3) tobacco use is a risk factor for six of the eight leading causes of death. According to estimates for 2005, 22% of adults worldwide currently smoke tobacco. Some 36% of men smoke compared to 8% of women. Nearly two-thirds of the world's smokers live in just 10 countries: Bangladesh, Brazil, China, Germany, India, Indonesia, Japan, the Russian Federation, Turkey, and the United States, which collectively comprise about 58% of the global population.

As per National Family Health Survey (NFHS 3),(4) 28.6% of men in Chennai smoke cigarettes or bidis.

Women and children living with smokers are at increased risk of premature death and disease from exposure to secondhand smoke. Interventions to protect women and children from household/public places, second hand smoke need to be strengthened.(5)

This necessitates the Central and State Governments in India to act, to curb this menace to protect the general public.

In this background, the air nicotine monitoring was conducted in Chennai with assistance from The Johns Hopkins Bloomberg School of Public Health, USA. With a objective.

- To measure levels of indoor air nicotine in public places (hospitals, schools, governmental offices, restaurants, and bars/nightclubs) in Chennai.
- To establish baseline levels of second hand smoke exposure to support more progressive smoke-free policies and to monitor and evaluate progress towards a smoke-free Chennai - project which aims to ensure smoke free public places in Chennai city through education and enforcement.

## Materials and Methods

### Overview of monitoring

The monitoring in selected buildings was done using passive monitors for vapor-phase nicotin. The parts of a vapor-phase nicotine passive monitor are

- Filter cassette: It is made with polystyrene (plastic). It has a clip in the superior part that will be very useful to correctly place the monitor. In the inferior part you can find a pad to support the filter.
- The filter treated with sodium bisulfate: It is able to keep the nicotine that is filtered from the environment. This filter will be carefully separated from the rest of the monitor in the lab, where the level of nicotine will be analyzed using gas-chromatography.
- The nucleopore windscreen: It protects the filter and lets the air pass through it with a flow rate equal to 0.024 L/min to allow the environmental nicotine to reach the filter. The size of the pores allows the passage of nicotine. The monitor weighs 16g.

The placement of air nicotine monitors in selected convenience sample of buildings [Table 1], with the consent from the building owners was done for measuring vapor-phase nicotine as per the technical guidance/protocol given by The Johns Hopkins Bloomberg School of Public Health.

**Table 1:** Number of buildings, number of monitors, location of the monitors, and the sampling time for each of the places selected in each city in the study

Places (number)	Location of monitors	Number of samples	Sampling time
Hospital (5)	Waiting room	1	1 week
	Doctor's lounge	1	
	Patient floors	2	
	Cafeteria	2	
		Subtotal: 30 (+6, 3 blanks and 3 duplicates)	
Secondary schools (5)	Cafeteria	2	1 week (Mon-Fri)
	Teachers' lounge	1	
	Students' bathroom (boys/girls)	2	
	Stairwells	1	
		Subtotal: 30 (+5, 3 blanks and 2 duplicates)	
City government (5)	Working offices	2	1 week (Mon-Fri)
	Lobby	1	
	Cafeteria	2	
	Bathrooms (male/female)	2	
		Subtotal: 35 (+6, 3 blanks and 2 duplicates)	
Restaurants (10)	Smoking area	1	1 week
	Non-smoking area (or main dining room if	1
	no restriction policy)		
		Subtotal: 20 (+4, 3 blanks and 3 duplicates)	
Entertainment [Bars/Clubs] (10)	Smoking area	1	1 week
	Non-smoking area (or main bar area if no restriction policy)	1	
Total (35)		Subtotal: 20 (+4, 2 blanks and 2 duplicates) 135 (+25 quality control)	

To ensure that all the common public places were included in the study, the monitors were placed in hospitals (patient waiting room, doctors room, cafeteria etc), schools (teachers room, cafeteria, students bathroom, steps, etc.), government buildings (lobby, cafeteria, bathrooms etc), restaurants (eating area, smoking and non-smoking area, etc.), and entertainment places like bar and clubs (eating and drinking area, smoking and non-smoking area, etc.).

The following guidance were used for placement of the monitors

- Hang the monitor in the air, 1-2 m from the floor.
- Hang monitors at least 1 m away from an open window or a ventilation system.
- Hang monitors at least 1 m away from a potential regular smoker.
- Do not hang monitors in an area where air does not circulate (i.e. “dead spots”), such as a corner, under a shelf, or on curtains.
- Ensure monitors are not too visible or accessible to avoid people tampering with them.
- Some good places include beams, nails or even plants or lamps.

The blank (10% of the total monitors) and duplicate (10% of the total monitors) monitors were placed to ensure quality control. Laboratory analysis is performed using gas chromatography.

All the air monitors was supplied free of cost by Johns Hopkins University through The UNION, (The International Union Against Tuberculosis and Lung Disease) along with technical know how. All the monitors were given unique coding to facilitate tracking and data analysis at Johns Hopkins University, where analysis was done free of cost for us. The study was conducted during June 2008.

### Results and Discussion

Out of the 160 monitors placed in 35 places, results from 71 air nicotine monitors from 25 buildings are reported here after excluding blanks, duplicates, erroneous, and ripped monitors.

A summary of the findings from this study are presented in Table 2. Which is similar to the findings in the other parts of the world.(6)

**Table 2:** Air nicotine concentrations (mg/m^3^) - Chennai

Building type	Number of buildings	Number of monitors	Median concentration (μg/m^3^)	Low (μg/m^3^)	High (μg/m^3^)
Entertainment	4	5	0.15	<LOD*	0.97
Government	5	27	0.16	<LOD*	0.77
Hospitals	5	16	0.19	<LOD*	0.60
Restaurants	6	6	0.60	0.39	8.47
Smoking area	-	2	4.53	0.61	8.47
Schools	5	17	0.22	0.03	0.61

* Concentration below limit of detection (LOD)

**Figure 1:**
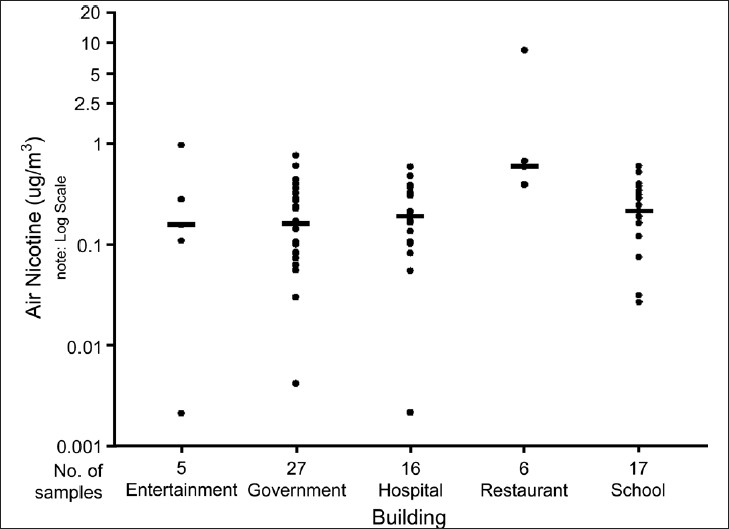
Each dot represents the air nicotine concentration for a single monitor. Some dots may overlap others in the figure. The bar reflects the median (50% percentile) air nicotine concentration for each venue. Values below the LOD (limit of detection) are treated as 1/2 of LOD; Air nicotine concentrations (mg/m^3^) - Chennai; Second-hand smoke monitoring: Chennai
- Similar air nicotine concentrations were observed in entertainment venues, schools, hospitals, and government offices.

The key findings of the study were:

- All monitors recorded detectable levels of air nicotine in restaurants and schools.
- In government buildings, hospitals, and entertainment venues, 96%, 94%, and 80% of air nicotine monitors, respectively, were above the detection limit.
- The highest median levels of air nicotine were found in restaurants [Table 2 and Figure 1].

### Data limitations

- These air nicotine concentrations will underestimate actual exposure in buildings that are unoccupied for a portion of the day as air concentrations represent 24-h integrated exposure over a 5- or 7-day period.
- These data provide a 1-week snapshot generated from a small sample of buildings and, therefore do not represent indoor air concentrations for the whole city.
- Monitors were placed in locations where people tend to congregate, not necessarily where the majority of smoking is occurring.
- Possibility of interference by others with the monitoring equipment may results in biased results

The finding of airborne nicotine in public places in Chennai provides a basis for enforcing smoke-free initiatives and for strengthening the protection of the public particularly children and workforce from unwanted exposure to secondhand smoke.
